# Differential Single-Crystal Waveguide Ultrasonic Temperature Measurements Based on Magnetostriction

**DOI:** 10.3390/mi16111274

**Published:** 2025-11-13

**Authors:** Yanlong Wei, Gang Yang, Gao Wang, Haijian Liang, Hui Qi, Xiaofang Mu, Zhen Tian, Fujiang Yuan, Qianxiang Zhang

**Affiliations:** 1 School of Computer Science and Technology, Taiyuan Normal University, Taiyuan 030619, China; 2 School of Information and Communication, North University of China, Taiyuan 030051, China; 3 State Key Laboratory of Dynamic Measurement Technology and School of Software, North University of China, Taiyuan 030051, China; 4 James Watt School of Engineering, University of Glasgow, Glasgow G12 8QQ, UK

**Keywords:** sapphire waveguide, differential principle, magnetostriction, high-temperature sensing

## Abstract

In extremely harsh high-temperature environments in aerospace, industrial manufacturing and other fields, traditional ultrasonic temperature measurement technology has certain limitations. This paper proposes a differential single crystal sapphire ultrasonic temperature measurement method based on the magnetostrictive effect. This method abandons the traditional sensitive flexural structure and uses two single-crystal sapphire waveguides of the same material, same diameter, and slightly different lengths as sensing elements. By measuring the time delay difference between their end-face echoes, the sound velocity is inverted and the temperature is measured. COMSOL multi-physics v6.1 simulation was used to optimize the bias magnetic field design of the magnetostrictive transducer, which improved the system’s energy conversion efficiency and high-temperature stability. Experimental results show that in the range of 300–1200 °C, the sensor delay increases monotonically with increasing temperature, the sound speed shows a downward trend, and the repeatability error is less than 5%; the differential processing method effectively suppresses common mode noise in the range of 300–700 °C, and still shows high sensitivity above 800 °C. This research offers a technical solution with high reliability and accuracy for temperature monitoring in extreme environments such as those characterized by high temperatures and high pressures.

## 1. Introduction

With the continuous development of contemporary industry and scientific research, ultrasonic technology is no longer limited to the classic non-destructive testing and clinical diagnosis categories, but has gradually evolved into a detection and control method with rich functions. At present, this technology is rapidly expanding into the frontier direction of multidisciplinary intersection, showing a wide range of application prospects. Specifically, the flexible wearable ultrasound device has successfully achieved continuous and real-time tracking of human physiological parameters, opening up a new way for personalized health management; the imaging strategy based on dual-frequency excitation and array detection has greatly improved the image clarity and signal quality of the internal structure of blood vessels. At the same time, with the rise in ultrasonic neurostimulation and sonogenetic tools, sound waves are also showing transformative potential in biomedical directions such as non-invasive treatment and visual function repair. The above achievements fully prove the diverse functions and strong environmental adaptability of ultrasonics as a type of physical tool, thus laying a theoretical and technical foundation for its in-depth application in emerging situations, such as extreme working conditions sensing.

In high-end industrial processes such as aerospace engine testing, liquid metal reactor operation, and solid rocket propellant combustion, accurate monitoring of ultra-high temperature conditions is a key link to evaluate system efficiency and ensure operational safety. Therefore, it is of great engineering value and scientific significance to develop temperature sensing technology that can work stably for a long time under harsh conditions such as high heat, high pressure, and strong corrosion.

To date, widely adopted traditional temperature measurement methods, such as thermocouples and infrared technology, exhibit limited performance under such extreme operating conditions. Specifically, thermocouple temperature measurement devices are prone to material degradation in oxidizing environments, leading to significant deviations in measurement results. Meanwhile, infrared thermometry enables non-contact measurement; however, its signals are susceptible to interference from multiple factors, including the composition of the measurement path medium, smoke, and contamination of the observation window, thus compromising the reliability of the final data [1,2].

In contrast, ultrasonic temperature measurement technology has attracted widespread attention due to its advantages of non-contact, wide measurement range, good stability, and suitability for harsh environments. However, existing ultrasonic temperature measurement methods usually rely on processing sensitive grooves on the sensor to achieve temperature calibration [3,4,5]. Currently, North China University uses single-crystal sapphire as an ultrasonic sensor. By processing sensitive grooves on the surface of the waveguide, the combined application of ultrasonic temperature measurement and single-crystal sapphire has been initially realized. However, to ensure that the echo signal has sufficient clarity and resolution, the sensitive flexure is usually designed to have a long length, which makes it difficult to ensure that the entire flexure remains in a temperature field; in addition, the groove depth of the sensitive flexure generally maintains 30% to 40% of the diameter of the waveguide rod, which causes the flexure to easily break during the calibration process [6]. It also causes energy loss at the node during ultrasonic energy transmission, which in turn brings errors to the final measurement results.

In response to the above problems, accurate temperature measurement faces further technical challenges. This research aims to propose and verify a differential single-crystal sapphire ultrasonic temperature measurement method based on the magnetostrictive effect. Its core innovation points include the following three aspects:

Structural innovation: Abandoning the traditional sensitive groove design, two single-crystal sapphire waveguides with slightly different lengths and consistent materials are used to achieve temperature inversion by measuring the end-face echo delay difference, effectively avoiding structural fragility and energy scattering problems.

Transducer optimization: Utilize the COMSOL Multiphysics simulation system to optimize the bias magnetic field configuration of the magnetostrictive transducer, thereby enhancing the system’s energy conversion efficiency and signal stability in high-temperature environments.

System integration and verification: Construct a complete differential ultrasonic excitation and signal processing system to achieve temperature measurement in the range of 300–1200 °C, verifying the effectiveness of the differential method in suppressing common mode noise and improving measurement sensitivity.

This research provides a technical path with reliable structure, high accuracy, and strong anti-interference ability for temperature monitoring in extreme environments.

## 2. Experimental Principle

According to the theory of thermoelastic dynamics, the propagation velocity of ultrasonic waves in solid media depends primarily on the material’s elastic modulus and density. When the internal temperature of the material changes, both of these parameters undergo corresponding alterations, leading to variations in the ultrasonic wave velocity. Therefore, the propagation velocity of ultrasonic waves in solids can be expressed as:

(1) $$v=\sqrt{\frac{E(T)}{\rho (T)}}$$

where v is the speed of sound; E is the material’s elastic modulus; ρ is the density; and T is the ambient temperature. Thus, the ambient temperature within the internal temperature field can be estimated by measuring the propagation speed of ultrasonic waves.

**Figure 1:**
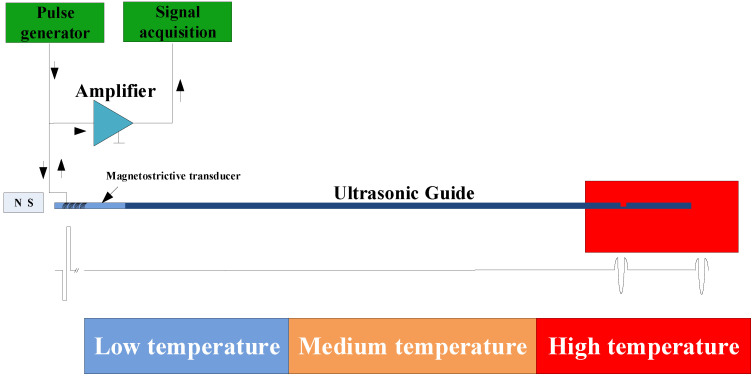
Principle of traditional ultrasonic temperature measurements.

In traditional ultrasonic temperature measurement methods, the temperature is determined by detecting the time delay difference between groove waves and end-face waves under different temperature conditions (Figure 1). The sensitive bend section serves as the temperature measurement segment; the length of this section must cover the target area and the material must meet temperature resistance and mechanical strength requirements. Typically, the measurement segment spans several wavelengths to ensure sufficient measurement space, as expressed in Equation (2):

(2) $$L_{IrRh}\geq m\lambda =m\frac{c}{f},m\approx 1∼2$$

Based on the ultrasonic frequency (250 kHz), the sensor’s sound velocity at room temperature, and relevant design parameters (e.g., m = 2), the sensitive bend length of the alloy waveguide rod was computed as 40 mm. This would result in significant temperature field variations during calibration experiments.

**Figure 2:**
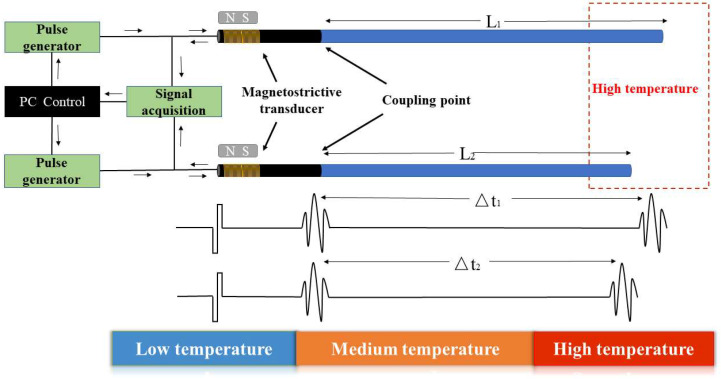
Schematic diagram of differential ultrasonic temperature measurement principle.

To overcome the aforementioned limitations, this study employs two waveguide rods consisting of identical material with the same diameter but slightly differing lengths. As shown in Figure 2, the proposed design requires no additional processing of the sensors. This approach avoids the shortcomings of traditional sensitive bending-element designs and extends the sensor’s service life. Additionally, ultrasonic waves are introduced into both sensors via a transducer. Acoustic waves generate echoes at both the coupling points and the end faces due to acoustic impedance mismatch.

When propagating along the $L_{1}$ acoustic waveguide, $\Delta t_{1}$ is:

(3) $$\Delta t_{1}=2\int_{0}^{L_{1}}\frac{1}{V(T)}\mathrm{dl}$$

When propagating along the $L_{2}$ acoustic waveguide, $\Delta t_{2}$ is:

(4) $$\Delta t_{2}=2\int_{0}^{L_{2}}\frac{1}{V(T)}\mathrm{dl}$$

The relative temperature delay of the temperature measurement sensitive intercept is to use the length difference in the two waveguide rods as a constant “sensitive temperature measurement intercept”. Since the two waveguide rods are in the same temperature field T, the sound speed v of ultrasonic waves propagating in them is the same. Therefore, the difference in length after the ultrasonic wave travels Δt is:

(5) $$\begin{array}{l} \Delta t=\Delta t_{1}-\Delta t_{2}\\ =2\int_{0}^{L_{1}}\frac{1}{V(T)}\mathrm{dl}-2\int_{0}^{L_{2}}\frac{1}{V(T)}\mathrm{dl}\\ =2\int_{0}^{L_{1}-L_{2}}\frac{1}{V(T)}\mathrm{dl} \end{array}$$

The relationship between the speed of sound v and the time delay Δt is given by:

(6) $$v(T)=\frac{2(L_{1}-L_{2})}{\Delta t}$$

The physical significance of this formula is that it converts the absolute sound speed v(T), which is difficult to accurately measure, into a measurement of the time difference Δt of two echoes. The length difference ΔL is a fixed geometric quantity, so the core advantage of the differential measurement principle is its excellent common-mode rejection. In an ultrasonic temperature measurement system, in addition to temperature, a variety of environmental factors and the inherent random noise of the system will cause the measured absolute delay $t_{1}$ and $t_{2}$ to slowly change or fluctuate. This type of interference can be collectively referred to as “thermal drift” or “common mode noise”.

In a single-sensor system, this drift will be directly misjudged as a temperature change, introducing measurement errors that are difficult to correct. In the differential configuration proposed in this study, since the two waveguide rods are in almost the same physical and electrical environment, most of the above interference factors will act on the two sensing channels simultaneously in a highly correlated form. Therefore, when calculating the difference between the two delays $\Delta t=\Delta t_{1}-\Delta t_{2}$ these common interference components are significantly canceled out.

## 3. Preparation of Sensors

### 3.1. Sensor Selection

In ultrasonic temperature measurement experiments, sensor design has a significant effect on measurement outcomes. Single-crystal optical fibers, which are crystalline materials combining a fiber structure with waveguide properties, have garnered extensive research attention in recent years [7,8,9]. Previous temperature measurement studies predominantly employed refractory metals as acoustic waveguide rods; however, these metals are prone to oxidation in high-temperature environments, which compromises sensor stability [10,11]. Single-crystal oxides can overcome this limitation [12]. Typically, ultrasonic temperature measurement schemes incorporate a sensitive notch in the sensor for temperature detection. This design reduces the structural integrity of the sensor by scattering ultrasonic energy at the notch and increasing susceptibility to fracture. Both of these phenomena adversely affect measurement accuracy.

The present study integrates two single-crystal oxide rod waveguides that are identical except for their slightly different lengths. By substituting node waves with surface waves at the end of the shorter waveguide rod, the proposed method prevents the structural fragility and energy scattering caused by grooves, thereby significantly enhancing the temperature measurement accuracy. Among established single-crystal oxides, sapphire exhibits the highest flexural strength (300–400 MPa) and resistance to fracture [13,14]. Thus, sapphire was selected as the sensor material for this study. The properties of sapphire are summarized in Table 1.

**Figure 3:**
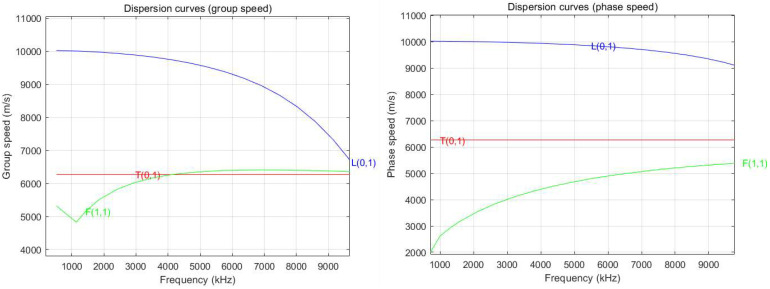
Dispersion curves of single-crystal sapphire.

The dispersion curve of sapphire was analyzed using Matlab R2018a code for guided wave analysis. As shown in Figure 3, variations in the waveguide rod diameter and frequency altered the group velocity and phase velocity. To minimize dispersion, a single-crystal waveguide rod with a diameter of 0.5 mm was employed.

In a differential ultrasonic temperature measurement system, the selection of the reference length L of the waveguide rod and its length difference ΔL is crucial, as it directly affects the temperature measurement sensitivity and signal-to-noise ratio of the system. According to the relationship between the speed of sound v, the length of the waveguide rod L and the ultrasonic propagation time Δt, the unit sensitivity of the sensor is defined as:

(7) $$S=\frac{\left|\Delta v\right|}{v_{avg}^{2}\Delta T}\times 10^{9},\Delta v=v(T_{2})-v(T_{1}),v_{avg}=\frac{v(T_{1})+V(T_{2})}{2},\Delta T=T_{2}-T_{1}$$

In the formula, Δv is the change in sound speed in the temperature interval, $v_{avg}$ is the average sound speed in the interval, and ΔT is the temperature span of the interval. This formula describes how sensitive the speed of sound is to changes in travel time caused by changes in temperature. The greater the sensitivity, the more significant the impact of small changes in temperature on the ultrasonic flight time. The premise for the establishment of the differential principle is that the two waveguide rods are in the same temperature field. Excessively large ΔL will cause the sensitive sections of the two waveguide rods to be spatially separated, which will introduce significant measurement errors in an environment where there is a temperature gradient.

Considering the size of the constant temperature zone of the laboratory high-temperature furnace (100 mm × 100 mm × 100 mm) and leaving a margin for installation, the reference length L was set to approximately 230 mm in this study. In order to ensure that the two waveguide rods can be completely placed in a uniform temperature field, the end positions of their effective sensitive sections should be as close as possible. Therefore, the choice of the length difference ΔL requires a balance between sensitivity and spatial constraints.

Based on the above analysis, this study selected the longer waveguide rod $L_{1}$ = 235.0 mm, the shorter waveguide rod $L_{2}$ = 228.9 mm, and the length difference ΔL = 6.1 mm. This design allows the change in the time delay difference Δt caused by temperature to reach the order of tens of microseconds in the measurement range of 300–1200 °C. It can be resolved with high precision by the acquisition system and ensures that the distance between the ends of the two sensors is extremely small. It perfectly meets the spatial constraints of the constant temperature zone of the high-temperature furnace and is an optimization result that takes into account measurement sensitivity and engineering feasibility.

### 3.2. Sensor Fabrication

Recently, significant progress has been made in terms of the preparation of single-crystal oxide materials. Researchers at the North China University of Technology and Shandong University have fabricated high-melting-point single-crystal oxide fibers comprising alumina and magnesia-alumina spinel, demonstrating major breakthroughs in material processing techniques [15]. Single-crystal oxides are typically grown using melt-growth methods, e.g., micro-drawing, pulling, and laser-heated substrate growth [16,17]. The laser-heated substrate method eliminates the need for crucibles, thereby reducing contamination risks while supporting rapid growth rates and easy diameter control. This approach enables the production of high-purity single-crystal oxides, which enhance the overall performance of single-crystal waveguides.

**Figure 4:**
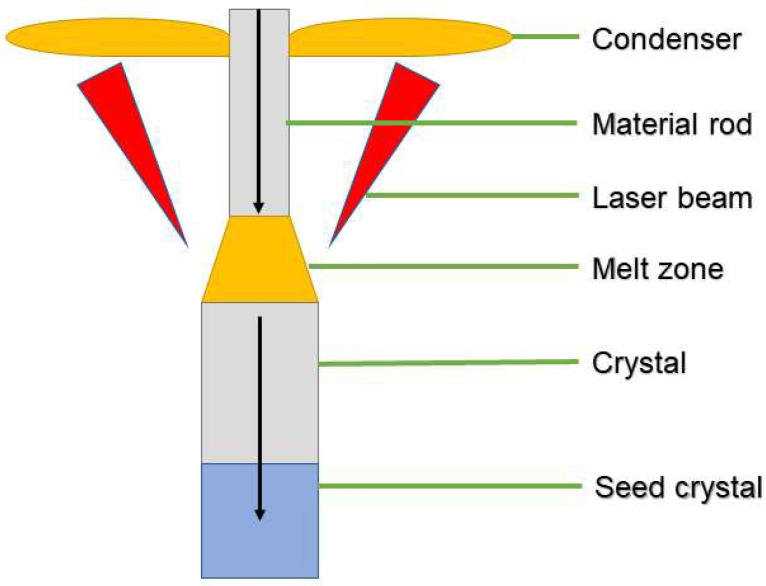
Sensor fabrication.

To obtain high-quality single-crystal waveguide rods, the relative positions of the feed rod and seed crystal must be adjusted, followed by a gradual increase in the CO_2_ laser output power (Figure 4). The laser beam passes through a ZnSe observation window into the crystal growth chamber, where it is converted into an annular spot by a conical mirror and focused onto the feed rod’s tip to generate a uniform hemispherical melt zone. Once the melt zone stabilizes, the seed crystal is lowered to come in contact with the melt, thus establishing a flat solid–liquid interface. Simultaneously, the pulling speed of the seed crystal and the feed rate of the ingot rod are precisely coordinated. Throughout the growth process, the laser power and focal position must remain constant to ensure a consistent waveguide diameter. This technique yields high-quality single-crystal waveguides of various sizes that meet the required specifications.

### 3.3. Preparation of Transducers

Ultrasonic excitation methods are generally divided into two types. One is a piezoelectric ultrasonic transducer. The piezoelectric ultrasonic transducer uses the piezoelectric effect of the internal piezoelectric chip to complete the mutual conversion between electrical signals and ultrasonic signals. It needs to be connected to the waveguide wire through a coupling agent to ensure that the ultrasonic waves can be effectively transmitted and complete the excitation and reception of ultrasonic waves. The other ultrasonic excitation method is a magnetostrictive transducer. The magnetostrictive transducer completes the excitation and reception of ultrasonic waves based on the magnetostrictive effect. Magnetostrictive transducers generally have high energy conversion efficiency and can achieve large power output with relatively small volume and weight. Based on the significant advantage of magnetostrictive transducers in high energy efficiency, magnetostrictive ultrasonic transducers were used in this experiment [18,19].

**Figure 5:**
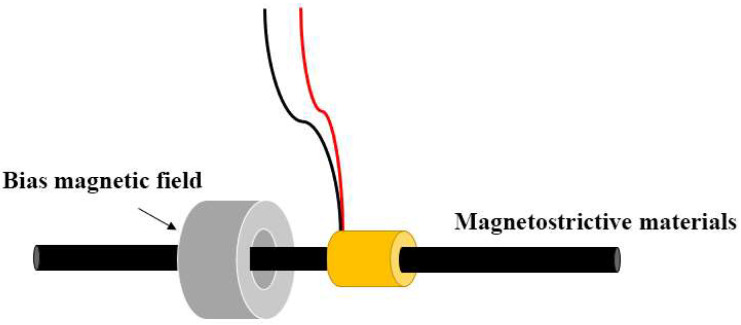
Magnetostrictive transducer (red and black lines represent the positive and negative poles).

As shown in Figure 5, the magnetostrictive transducer is mainly composed of three parts: magnetostrictive material, induction coil and bias magnetic field. The transducer design used in this study integrates excitation and reception functions into a single coil, achieving the integration of ultrasonic transmission and reception. In this system, the introduction of a bias magnetic field plays a key role. It not only establishes a stable static operating point for the transducer and suppresses frequency doubling interference that may occur during ultrasonic wave propagation, but also helps to significantly improve its energy conversion efficiency. In order to further systematically explore the impact of bias magnetic field strength on transducer performance, this paper conducts numerical simulation and calculation analysis on the relationship between the magnetic properties of magnetostrictive materials and the bias magnetic field based on the COMSOL Multiphysics multi-physics simulation platform.

A ring-shaped neodymium iron boron permanent magnet (external dimensions: 10 × 10 mm; inner diameter: 4 mm) was used for the bias magnetic field to optimize the magnetic domain alignment efficiency of the magnetostrictive transducer. This magnet has a high magnetic flux density and meets high-temperature operational requirements. COMSOL simulation software was used to simulate the magnetization capabilities and magnetostrictive effect of the device. This process identified the optimal position for the permanent magnet relative to the magnetic flux density and revealed how this flux density is affected by temperature.

**Figure 6:**
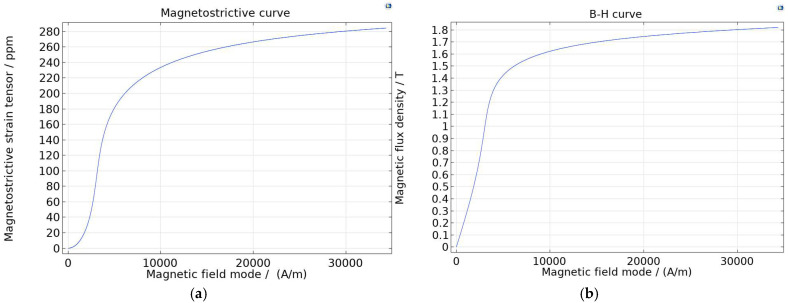
Magnetostrictive material characteristic Curves: (**a**) magnetostrictive curve; (**b**) current density-magnetic flux curve.

As shown in Figure 6, the magnetic flux density and magnetostriction effect change with changes in the magnetic field modulus. In magnetostrictive materials, a higher rate of change in magnetic flux density indicates a more pronounced magnetostrictive effect. Herein, the magnetostrictive effect is optimal when the magnetic flux density of the bias field reaches 0.9 T.

**Figure 7:**
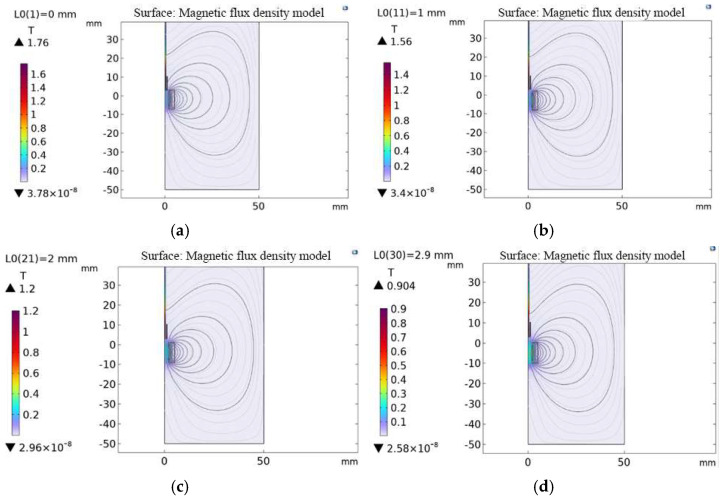
Optimal position of the permanent magnet: models of magnetic flux density at distances of (**a**) 0, (**b**) 1, (**c**) 2, and (**d**) 2.9 mm.

A parametric scan of the permanent magnet position identified the location yielding an optimal magnetic flux density of approximately 0.9 T. The simulation results shown in Figure 7 indicate that when the permanent magnet is 2.9 mm from the ferrite alloy, the magnetic flux density reaches 0.904 T, at which point, the transducer efficiency is maximized.

**Figure 8:**
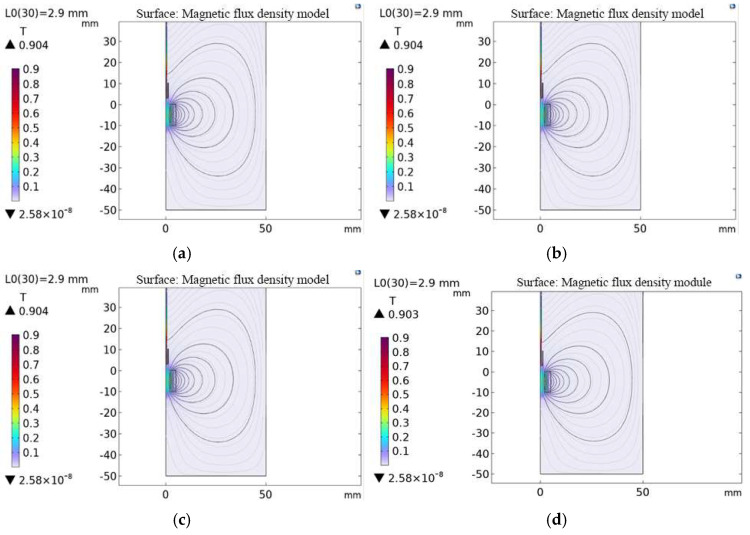
Magnetic flux density at (**a**) 200 °C, (**b**) 300 °C, (**c**) 400 °C and (**d**) 500 °C.

**Figure 9:**
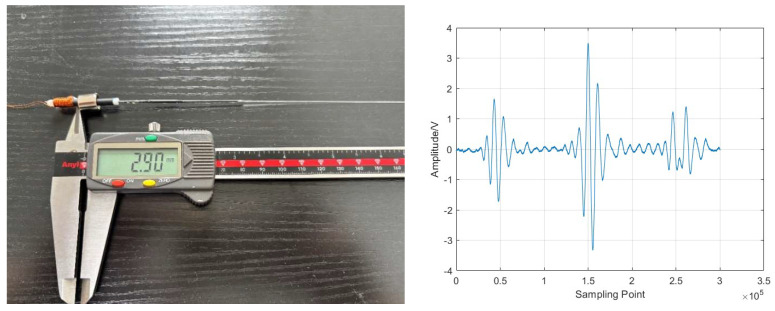
Magnetostrictive transducer preparation results.

During the ultrasonic temperature measurement calibration experiment, although the iron–gallium alloy was placed outside the calibration furnace, due to the thermal conductivity of the material itself, changes in ambient temperature would also cause it to rise significantly and steadily. In order to systematically quantify the impact of temperature on the magnetostrictive characteristics, the magnetic flux density of the magnetostrictive material at room temperature to 600 °C was analyzed through simulation experiments. As shown in Figure 8 and Figure 9, the simulation experiment results show that an increase in temperature will cause the magnetic domain orientation energy barrier of the iron–gallium alloy to decrease, causing the saturation magnetostriction coefficient to attenuate, confirming the strong correlation between temperature and the magnetostrictive effect. However, in this experiment, the magnetostrictive device was placed outside the temperature field. Within the range of the excitation magnetic field, the fluctuation amplitude of the temperature-induced magnetic flux density was very small. This change was significantly lower than the 0.5% full-scale accuracy threshold of the Hall sensor. Therefore, it will not cause substantial interference when collecting experimental data.

In the final magnetostrictive transducer (Figure 9), the bias magnetic field is 2.9 mm from the sensing coil. The coil has a total of 41 turns, and the single-crystal sapphire waveguide is bonded to the iron–gallium alloy transducer material using casting adhesive. It has been confirmed by experiments with the data collection system that under this configuration, the echo signal has extremely high discrimination, the waveform is clear, and it is easy to extract. Very helpful for data collection.

## 4. Experiment Details

### 4.1. Differential Ultrasonic Excitation System

**Figure 10:**
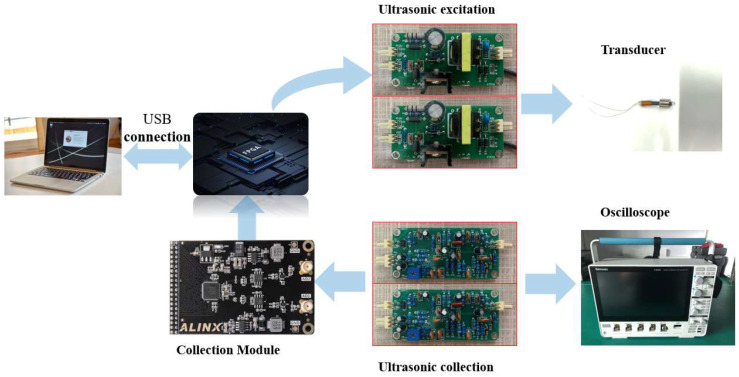
Differential ultrasonic temperature measurement architecture.

The system architecture is illustrated in Figure 10. An FPGA serves as the main control chip, receiving commands from the host computer software via a USB module. It generates a 250 kHz trigger pulse, which is impedance-optimized by a power amplifier before being applied to the transducer. The transducer then converts the electrical signal into an ultrasonic signal, which propagates along the sapphire waveguide rod, generating reflected echoes at the coupling point and the end face. The transducer converts these echo signals back into electrical signals, which are processed through a bandpass filter circuit to remove noise. Meanwhile, a gain adjustment circuit dynamically modulates the signal amplitude. The digital acquisition module transmits the filtered and amplified electrical signals via a USB module to the temperature measurement software, which stores and displays the collected data. Mutual correlation algorithms compute the time delay differences for both the end-face waves and the coupling waves, yielding the dual-sensor time delay difference. An external oscilloscope synchronously captures raw signals, thereby leveraging its high sampling frequency to enhance data precision.

**Figure 11:**
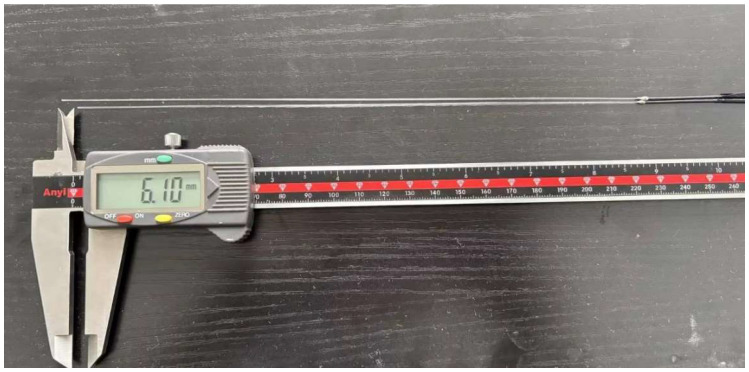
Single-crystal sapphire waveguide rods.

One sapphire waveguide rod was 235 mm in length and the other was 228.9 mm in length (Figure 11). After drilling a hole at one end of the magnetostrictive material, the single-crystal sapphire was inserted into the hole and the two materials were bound together using casting adhesive.

### 4.2. Experiment Setup

**Figure 12:**
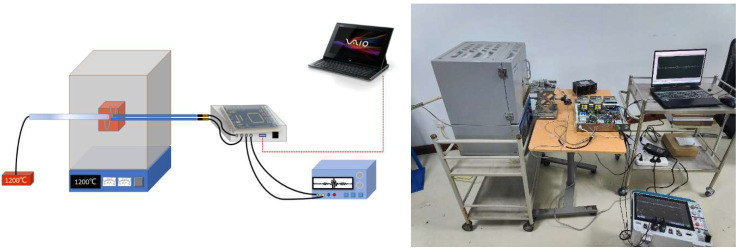
Experimental calibration setup.

As shown in Figure 12, the ultrasonic temperature measurement system employed in this study comprised a high-temperature experimental furnace, an ultrasonic temperature measurement device, ultrasonic transducers, and single-crystal sapphire components. During the calibration, the high-temperature furnace was gradually heated from room temperature to 1200 °C in 100 °C increments. After reaching each temperature point, it was held constant for 8 minutes. This program ensured uniform and stable temperature distribution within the furnace and full thermal equilibrium with the sensors. When installing the sapphire waveguide rod, the sensor’s sensitive end was positioned within a constant-temperature zone (100 × 100 × 100 mm) formed by heating with a silicon-molybdenum rod. It was then arranged parallel to a Type B standard thermocouple (accuracy = 0.30%). The accuracy and reliability of the final ultrasonic temperature measurement system were verified upon obtaining reliable temperature reference values [20].

## 5. Data Processing and Analysis

### 5.1. Data Processing

**Figure 13:**
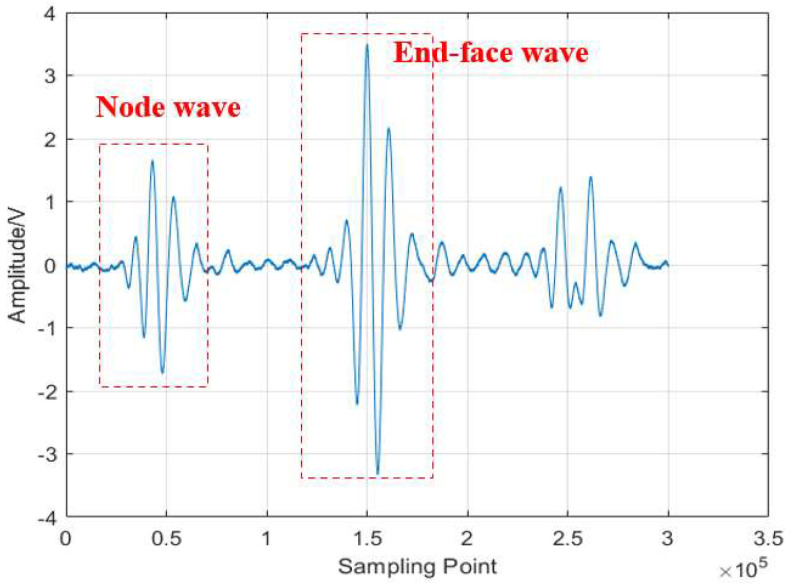
Cross-correlation algorithm to extract data.

After the temperature stabilizes, the ultrasonic echo signal is collected, the cross-correlation algorithm is used to calculate the sound wave flight time, and the corresponding sound speed is deduced from this. As shown in Figure 13, the original echo signal is first band-pass filtered and normalized to eliminate noise and unify the signal amplitude; then, a time window containing valid information is selected, and a cross-correlation operation is performed between the sensor node echo and the terminal reflection echo within the window. By identifying the peak position of the cross-correlation function, the time delay difference between the two echoes can be accurately obtained, and based on this, the sound speed in the material can be further inverted.

Although the amplitude of the echo signal will attenuate as the temperature increases, there is still a good correlation between the node wave and the end face echo. The cross-correlation algorithm can effectively evaluate the similarity between two waveforms to accurately determine the propagation time of ultrasonic waves [21]. In order to eliminate the influence of interference signals on the analysis results, when constructing the cross-correlation sequence, the time interval before the starting point of the node wave and after the end point of the terminal echo is selected as the calculation benchmark. This method can adaptively adjust the time window range and improve the robustness of time delay extraction.

### 5.2. Discussion on Sensor Structure and Long-Term Stability of Transducer

Although this study did not conduct a dedicated accelerated aging life test, the repeatability data obtained during the entire high-temperature calibration experiment (from room temperature to 1200 °C and multiple thermal cycles) can provide strong indirect evidence for the structural stability of the sensor.

**Figure 14:**
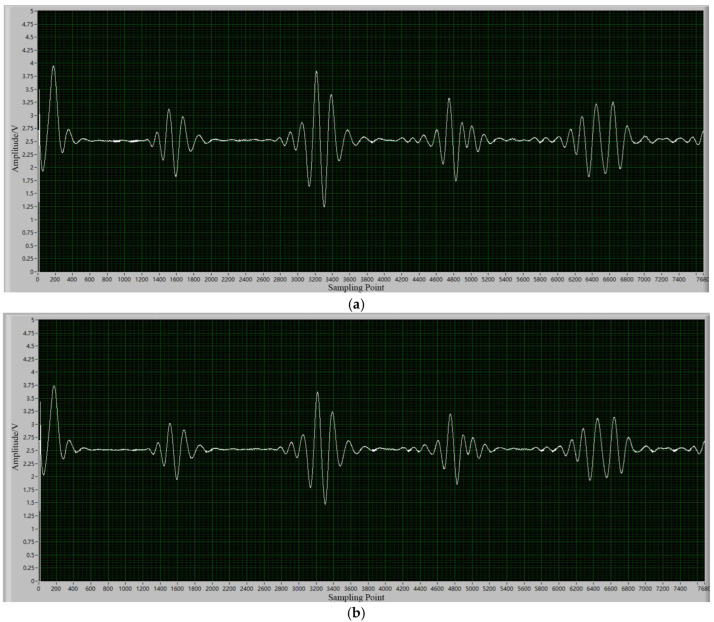
Real-time interface of data acquisition system under different temperatures: (**a**) 20 °C, (**b**) 1000 °C.

The magnetostrictive transducer and the single-crystal sapphire waveguide are bonded through industrial caster glue to achieve tight coupling between the two. During preparation, a sapphire waveguide with a diameter of 0.5 mm was embedded into an iron–gallium alloy rod with the same hole diameter preset at one end and fixed with caster glue. Although the upper limit of the long-term temperature resistance of the caster glue used is 280 °C, since the transducer assembly is installed outside the furnace, its actual operating temperature is lower than 200 °C, thus ensuring the stable performance of the bonding material. Direct evidence demonstrating the reliability of the interface comes from the quality of the echo signal: as shown in Figure 14, the amplitude and waveform characteristics of the coupling point echo are highly consistent at room temperature and 1000 °C. This phenomenon strongly depends on the stability of the interface acoustic impedance. The echo signal was slightly attenuated but did not affect the final result, indicating that the bonding interface had not been degraded or damaged, confirming that the structure remained stable and reliable throughout the experiment.

### 5.3. Data Analysis

**Figure 15:**
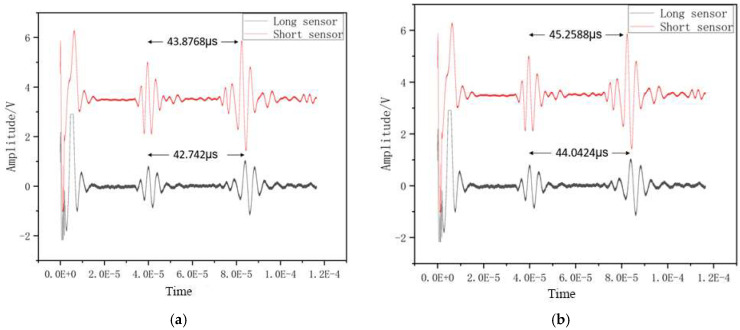
Ultrasonic echo signal acquisition and processing at (**a**) 20 °C and (**b**) 1000 °C.

This experiment aims to evaluate the performance of the differential single-crystal sapphire ultrasonic temperature measurement method based on the magnetostrictive effect. The test is carried out in a high-temperature calibration furnace, using an ultrasonic temperature measurement system to apply excitation and collect echo signals. As shown in Figure 15, the acoustic wave propagation time is obtained by collecting the peak moment of the excitation pulse at the coupling point and the terminal echo signal. In order to eliminate noise interference and improve temperature measurement accuracy, this experiment uses the differential principle to process data. Direct calculation of the delay difference between the end faces is unreliable. It has been experimentally verified that the coupling point delay changes significantly with temperature. Therefore, the optimization plan is to first calculate the time delay from the end face of each sensor to the coupling point, and then derive the sound speed from the difference. In order to examine the measurement repeatability, all experiments were conducted three times independently, focusing on analyzing the changes in time delay and sound speed with temperature and the effectiveness of the differential method in actual temperature measurement.

In ultrasonic temperature measurement systems, time delay analysis is a critical processing step to enable high-precision temperature inversion. The system developed in this study leverages the fact that the sound waves’ transit time through waveguide media varies with temperature. Specifically, accurate temperature information about the target environment can be acquired by precisely measuring the time delay of sound wave propagation. In differential ultrasonic temperature measurement configurations, mutual correlation algorithms are typically employed to separately extract the time delay differences between the end-face echoes and the coupled echoes from the two sensors, thereby deriving the differential time delay difference.

**Figure 16:**
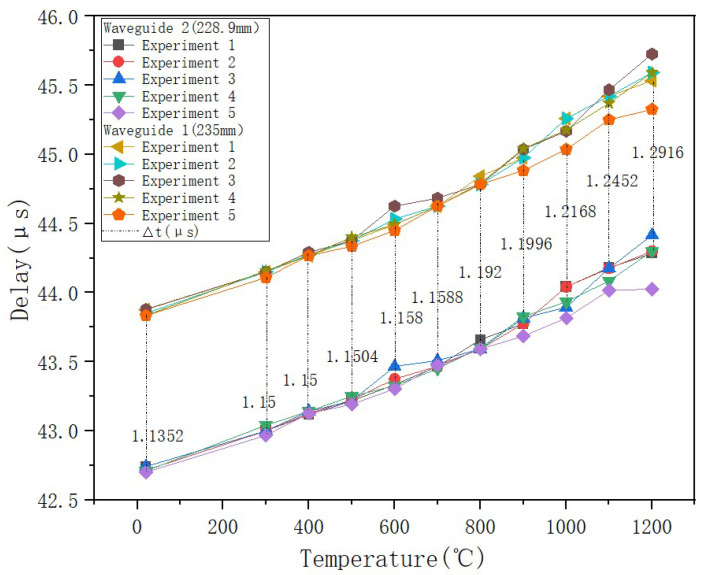
Delay as a function of temperature.

The time $\Delta t_{1}$ required for the ultrasonic wave to propagate along sensor $L_{1}$ is (Table 2):

The time $\Delta t_{2}$ required for the ultrasonic wave to propagate along sensor $L_{2}$ is (Table 3):

A significant upward trend in the time delay difference between the two sensing waveguide rods was observed. As the ambient temperature increased, the time required for ultrasonic waves to propagate from the coupling position to the end increased nearly linearly. The time delay also showed a monotonically increasing trend overall with increasing temperature (Figure 16). Between room temperature and 700 °C, the increase in time delay was moderate. However, when the temperature exceeded 800 °C, the rate of increase became markedly higher, reflecting the heightened sensitivity of the material’s acoustic properties to temperature at high temperatures. Three repeated experiments demonstrated the sensors’ excellent stability. However, data reproducibility had some fluctuation during the high-temperature phase. This phenomenon primarily stems from limitations in controlling temperature field uniformity in the high-temperature furnace, rather than inherent sensor performance.

**Figure 17:**
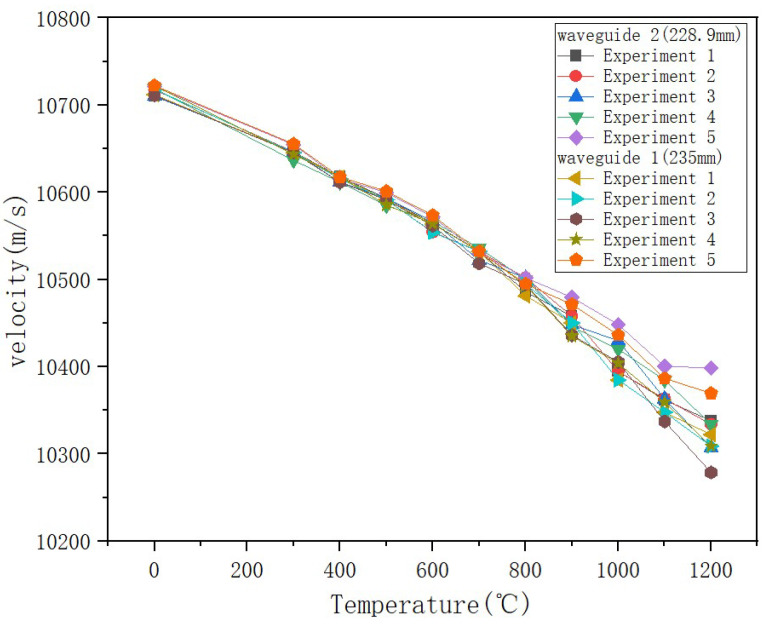
Sound velocity as a function of temperature.

To evaluate the thermal stability of the sensor, it is also necessary to analyze the sensor’s sound velocity experimental data. As shown in Figure 16 and Figure 17. The results show that during the temperature rise process, the delay between the end-face echoes of the two sensors and the coupling point gradually increases, and the sound speed shows a downward trend. This change is consistent with the known law of sound speed changing with temperature, indicating that the selected sensor can be suitable for ultra-high temperature experimental environments. Based on the peak information of the echo signal, a cross-correlation algorithm is used to calculate the time difference and sound speed value corresponding to each temperature point. The specific experimental data are as follows (Table 4 and Table 5):

The time delays of both sensors increased linearly with increasing temperature, while the sound velocity decreased. Both parameters exhibited good repeatability, consistent with thermoelastic theory [22,23]. These results demonstrate that the sensors maintained excellent structural stability in high-temperature environments. However, the difference in sound velocity between the two sensors widened as the temperature increased, reflecting an intensified temperature gradient.

The repeatability of a sensor reflects the upper limit of the random error of its measurement results, which refers to the maximum repeatability measured at all measurement points. Usually expressed as a percentage of the sensor’s full-scale output. The calculation formula is as follows:

(8) $$\xi_{R}=\frac{\Delta R}{Y_{FS}}\times 100\%$$

ΔR is the maximum deviation value of multiple measurements at the same temperature point. $Y_{FS}$ is the full-scale output value of the sensor. is the maximum change in delay over the entire temperature range. Just calculate each temperature point in the paper, and the repeatability is less than 5%.

The time difference Δt between the end-face wave and the coupled wave can be extracted from the experimental data using a cross-correlation algorithm and then substituted into Equation (6) to calculate the sound velocity. Herein, $L_{1}-L_{2}=6.1\mathrm{mm}$ represents the length difference. This approach effectively suppresses environmental noise and system drift.

The computed results are shown in Table 6.

**Figure 18:**
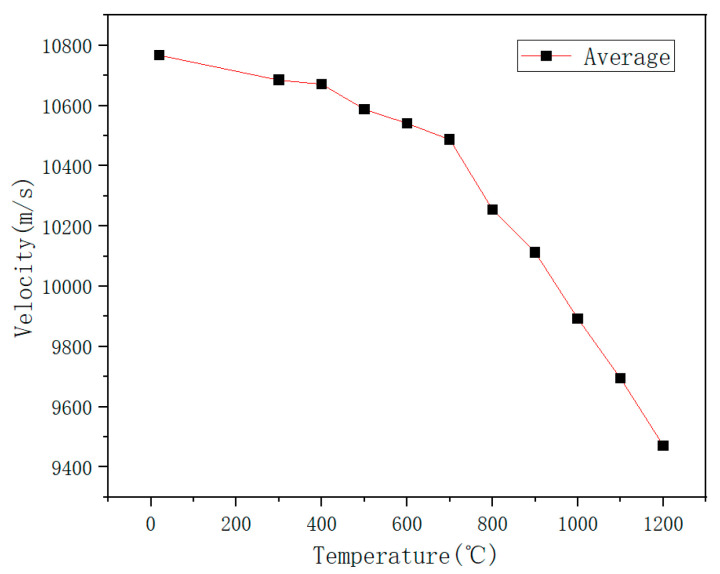
Sound velocity (averages) as a function of temperature.

The average of three independent experimental measurements at each datapoint was calculated to derive the curve showing the relationship between sound velocity and temperature (Figure 18). This method mitigates the interference of random errors in individual measurements, resulting in a curve that more reliably reflects the intrinsic acoustic properties of materials under high-temperature conditions. According to classical thermoelastic theory, both the Young’s modulus and the density of materials decrease with increasing temperature, leading to a corresponding reduction in the propagation speed of sound waves.

Based on the above experimental results, a comparative analysis of the sound speed data of the two sensors shows that the sound speed values measured by the two sensors are highly consistent in the range from room temperature to 700 °C, and the average change curve shows a linear pattern, indicating that the sensor is heated evenly in this temperature range, and the differential structure effectively offsets the common mode interference, reflecting the consistency of the temperature field distribution. However, when the temperature rises above 800 °C, although both sensors maintain stable operation, the sound velocity trend decreases steadily, and there is a difference in the numerical difference in sound velocity between the two sensors. This phenomenon shows that under high temperature conditions, although the entire experimental system is at the set temperature, the actual local temperatures of the two sensors are significantly different due to the actual axial and radial temperature gradients formed in the calibration furnace. On the one hand, this result verifies that the sensor itself still has good response consistency and structural stability under extremely high temperatures. The introduction of differential can greatly overcome the noise interference in the experimental environment; on the other hand, it also reveals that in current differential experiments, temperature field uniformity has become a key limiting factor affecting measurement consistency. In addition, the differentiation of sound speed data in high-temperature areas further demonstrates that the differential method proposed in this article has extremely high sensitivity in actual measurements and can effectively identify small and real temperature gradients, providing a reliable sensing basis for application in industrial scenarios with uneven actual temperature distribution.

Experimental data show that as the temperature increases, the time delay of a single sensor increases linearly, the sound speed decreases steadily, and the repeatability error is less than 5%, proving that sapphire material has excellent high-temperature performance. However, after the temperature exceeds 800 °C, the sound speed between the two sensors fluctuates greatly, indicating that there is an obvious temperature field gradient distribution in the experimental environment, which intensifies as the temperature increases. After the introduction of the differential principle, the system showed good stability in the temperature range of 300 °C to 700 °C, and the fluctuation range of the three experimental data was small.

When the temperature continues to rise above 800°C, the sound speed after differential processing decreases more significantly than the single sensor data. From the data analysis in Table 2, Table 3, Table 4 and Table 5, it can be seen that the sound speed of the two sensors in the same temperature field at the same time should remain consistent, but as the temperature rises, the sound speed difference between the sensors is different. As shown in the experimental layout in Figure 12, although the two waveguide rods are placed in parallel, there is a longitudinal spacing of about 6.1 mm at their ends. At high temperatures, both the axial and radial thermal gradients in the furnace may be intensified. At 1200°C, the sound speed drops 8% more than that of a single sensor. Since there is a temperature gradient in the temperature field, the differential data fluctuates greatly, which is due to the difference in temperature of the temperature measurement environment.

Experiments have confirmed that under constant temperature conditions, the differential scheme can greatly suppress noise interference and demonstrate extremely strong sensitivity. However, the limitation of current equipment is that it cannot accurately control the temperature distribution in the furnace, causing small deviations to be amplified and affecting the accuracy of differential measurements. But overall, applying the differential principle to ultrasonic temperature measurement solutions shows extremely high sensitivity and has good feasibility and application potential. 

### 5.4. An In-Depth Discussion of Differential Signal Fluctuations at High Temperatures

**Figure 19:**
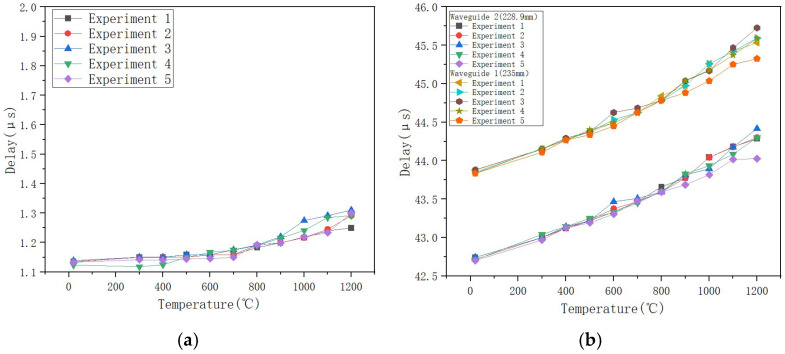
Single Sensor-Differential Output Comparison at (**a**) Differential, (**b**) Single Sensor.

In order to evaluate the attenuation effect of the differential structure on common-mode noise, this article conducts a comparative analysis of the output signal stability of a single sensor and a differential system under the same temperature field conditions. As shown in Figure 19, in the range from normal temperature to 300 °C, the delay measurement data of a single sensor shows significant fluctuations due to common mode factors such as power supply fluctuations and electromagnetic interference. Under the same experimental conditions, the fluctuation amplitude of the differential output is significantly reduced, indicating that the differential configuration can effectively compensate for the impact of common-mode interference. When the temperature rises above 800 °C, the fluctuation of the differential data increases due to the increase in the temperature field gradient, but the overall structure still exhibits better anti-interference characteristics than the single-ended structure.

Compared to previously reported ultrasonic temperature measurement approaches, the differential method developed herein mitigates the risks of node energy dispersion and structural fractures, thereby enhancing reliability and service life. By employing the principle of differential subtraction while maintaining relative temperature field stability, this approach suppresses noise interference during ultrasonic wave propagation [24]. Moreover, the absence of phase differences improves experimental precision.

## 6. Conclusions

This study successfully designed and verified a differential single-crystal sapphire ultrasonic temperature measurement system based on the magnetostrictive effect. This solution uses two sapphire waveguide rods with slightly different lengths to replace the traditional mechanically sensitive groove structure, effectively overcoming the problems of structural fragility and ultrasonic energy scattering, and significantly improving the structural integrity and signal reliability of the sensor in high-temperature environments. The optimized bias magnetic field design assisted by COMSOL multi-physics simulation further ensures the efficient and stable operation of the magnetostrictive transducer in a wide temperature range.

Experimental results show that the system has stable performance in the range of 300–1200 °C. The test sensitivity for temperature is extremely high, and the repeatability error is less than 5%. It can effectively suppress common-mode noise, especially at 300~700 °C. Although the overall performance of the system is excellent, in the high temperature area above 800 °C, the uniformity of the temperature field in the calibration furnace is difficult to accurately control, resulting in certain fluctuations in the differential sound velocity data. This reveals that the main limiting factor in current system performance is not the sensor itself, but the limits of the experimental environment that currently exists.

Facing the future, research work can continue to deepen from the following aspects: First, focus on improving the temperature field design to ensure a highly uniform and stable temperature distribution throughout the entire calibration range. This is the key to further improving the measurement accuracy and repeatability of the high-temperature section. Second, explore the reliability of differential data at higher temperatures. It has laid a solid foundation for the development of next-generation high-reliability high-temperature sensing technology.

## Figures and Tables

**Table 1:** Physical parameters of sapphire.

Density (g·cm^−3^)	Bending Hardness (MPa)	Young’s Modulus (GPa)	Melting Point (°C)	Thermal Conductivity (W·m^−1^ K^−1^)	Thermal Expansion Coefficient (K)
3.98	300–400	300–400	2053	35	5.8 × 10^−6^

**Table 2:** Delay differences for waveguide 1 (235 mm).

Temperature (°C)	Time Delay (μs)				
	First Experiment	Second Experiment	Third Experiment	Fourth Experiment	Fifth Experiment
20	43.8768	43.8512	43.88	43.834	43.834
300	44.1504	44.1504	44.1508	44.1588	44.1092
400	44.2676	44.2756	44.292	44.2664	44.2664
500	44.3756	44.3756	44.3756	44.4008	44.3352
600	44.4924	44.5336	44.6252	44.4924	44.4504
700	44.6252	44.6256	44.6836	44.625	44.6256
800	44.8424	44.784	44.784	44.7904	44.784
900	44.9748	44.9748	45.0364	45.0412	44.8836
1000	45.2588	45.2588	45.1684	45.1756	45.036
1100	45.4208	45.4208	45.4672	45.37	45.2504
1200	45.5336	45.592	45.726	45.592	45.3256

**Table 3:** Delay differences for waveguide 2 (228.9 mm).

Temperature (°C)	Time Delay (μs)				
	First Experiment	Second Experiment	Third Experiment	Fourth Experiment	Fifth Experiment
20	42.742	42.716	42.7412	42.7104	42.7008
300	43.0004	43.0004	43.0004	43.0408	42.9676
400	43.1168	43.1256	43.1416	43.1416	43.1256
500	43.2172	43.2252	43.2172	43.2508	43.1908
600	43.3344	43.3756	43.4664	43.3256	43.3048
700	43.4664	43.4668	43.5084	43.4508	43.4756
800	43.6596	43.592	43.592	43.6	43.592
900	43.7752	43.7752	43.8164	43.826	43.6852
1000	44.0424	44.042	43.8936	43.9344	43.8164
1100	44.1808	44.1756	44.1756	44.085	44.0172
1200	44.284	44.3004	44.416	44.3004	44.0256

**Table 4:** Sound velocity data for waveguide 1 (235 mm).

Temperature (°C)	Speed of Sound (m/s)				
	First Experiment	Second Experiment	Third Experiment	Fourth Experiment	Fifth Experiment
20	10,711.81125	10,718.06473	10,711.03008	10,722.27038	10,722.27038
300	10,645.43017	10,645.43017	10,645.33372	10,643.40516	10,655.37348
400	10,617.24602	10,615.32763	10,611.39709	10,617.53384	10,617.53384
500	10,591.40609	10,591.40609	10,591.40609	10,585.39486	10,601.0574
600	10,563.60187	10,553.82902	10,561.70282	10,563.60187	10,573.58314
700	10,532.16568	10,532.07128	10,518.40049	10,532.21289	10,532.07128
800	10,481.15177	10,494.81958	10,494.81958	10,493.32	10,494.81958
900	10,450.29661	10,450.29661	10,436.00288	10,434.89072	10,471.5308
1000	10,384.72076	10,384.72076	10,405.50473	10,403.84632	10,436.09557
1100	10,347.68212	10,347.68212	10,337.12215	10,359.26824	10,386.64852
1200	10,322.04789	10,320.14404	10,278.6161	10,308.82611	10,369.41596

**Table 5:** Sound velocity data for waveguide 2 (228.9 mm).

Temperature (°C)	Speed of Sound (m/s)				
	First Experiment	Second Experiment	Third Experiment	Fourth Experiment	Fifth Experiment
20	10,710.77629	10,717.29563	10,709.97445	10,718.70083	10,721.11061
300	10,646.41259	10,646.41259	10,646.41259	10,636.4194	10,654.5397
400	10,617.67107	10,615.50448	10,611.56749	10,611.56749	10,615.50448
500	10,593.00464	10,591.04411	10,593.00464	10,584.77531	10,599.47952
600	10,564.35534	10,554.32086	10,566.5011	10,566.5011	10,571.57636
700	10,532.2732	10,532.17628	10,522.10608	10,536.05457	10,530.04444
800	10,485.66638	10,501.92696	10,501.92696	10,500	10,501.92696
900	10,457.97621	10,457.97621	10,448.1427	10,445.85406	10,479.52167
1000	10,394.52891	10,394.62331	10,429.76653	10,420.08085	10,448.1427
1100	10,361.96719	10,363.18692	10,363.18692	10,384.48452	10,400.47981
1200	10,337.81953	10,333.99247	10,307.09654	10,333.99247	10,398.49542

**Table 6:** Experimental calibration data.

Temperature (°C)	Speed of Sound (m/s)				
	First Experiment	Second Experiment	Third Experiment	Fourth Experiment	Fifth Experiment
20	10,750.79309	10,747.00493	10,750.79309	10,857.95657	10,765.97247
300	10,608.69565	10,608.69565	10,605.00695	10,912.34347	10,686.75543
400	10,601.32082	10,608.69565	10,605.00695	10,846.37269	10,694.24965
500	10,531.76796	10,605.00695	10,531.76796	10,608.69565	10,660.60818
600	10,535.40587	10,535.40587	10,384.74634	10,455.94789	10,649.44134
700	10,528.13255	10,528.13255	10,381.21171	10,390.0528	10,608.69565
800	10,314.50795	10,234.89933	10,234.89933	10,248.65591	10,234.89933
900	10,170.05669	10,170.05669	10,000	10,039.49967	10,180.24032
1000	10,029.59553	10,026.29849	9570.128648	9829.197551	10,003.27976
1100	9838.709677	9797.622872	9445.648808	9494.163424	9892.961401
1200	9763.1242	9445.648808	9312.977099	9445.648808	9384.615385

## Data Availability

The data presented in this study are available on request from the corresponding author.
